# Combination of Smartphone MEMS Sensors and Environmental Prior Information for Pedestrian Indoor Positioning

**DOI:** 10.3390/s20082263

**Published:** 2020-04-16

**Authors:** Lu Huang, Hongsheng Li, Baoguo Yu, Xingli Gan, Boyuan Wang, Yaning Li, Ruihui Zhu

**Affiliations:** 1 College of Instrumental Science and Engineering, Southeast University, Nanjing 210018, China; hlcetc54@163.com; 2 State Key Laboratory of Satellite Navigation System and Equipment Technology, Shijiazhuang 050081, China; yubg@sina.cn (B.Y.); ganxingli@163.com (X.G.); boyuan@hrbeu.edu.cn (B.W.); 15631149037@163.com (Y.L.); zhu_lucking@163.com (R.Z.); 3 The 54th Research Institute of China Electronics Technology Group Corporation, Shijiazhuang 050081, China

**Keywords:** indoor localization, smartphone, dead reckoning, sensors, deep neural network

## Abstract

In view of the inability of Global Navigation Satellite System (GNSS) to provide accurate indoor positioning services and the growing demand for location-based services, indoor positioning has become one of the most attractive research areas. Moreover, with the improvement of the smartphone hardware level, the rapid development of deep learning applications on mobile terminals has been promoted. Therefore, this paper borrows relevant ideas to transform indoor positioning problems into problems that can be solved by artificial intelligence algorithms. First, this article reviews the current mainstream pedestrian dead reckoning (PDR) optimization and improvement methods, and based on this, uses the micro-electromechanical systems (MEMS) sensor on a smartphone to achieve better step detection, stride length estimation, and heading estimation modules. In the real environment, an indoor continuous positioning system based on a smartphone is implemented. Then, in order to solve the problem that the PDR algorithm has accumulated errors for a long time, a calibration method is proposed without the need to deploy any additional equipment. An indoor turning point feature detection model based on deep neural network is designed, and the accuracy of turning point detection is 98%. Then, the particle filter algorithm is used to fuse the detected turning point and the PDR positioning result, thereby realizing lightweight cumulative error calibration. In two different experimental environments, the performance of the proposed algorithm and the commonly used localization algorithm are compared through a large number of experiments. In a small-scale indoor office environment, the average positioning accuracy of the algorithm is 0.14 m, and the error less than 1 m is 100%. In a large-scale conference hall environment, the average positioning accuracy of the algorithm is 1.29 m, and 65% of the positioning errors are less than 1.50 m which verifies the effectiveness of the proposed algorithm. The simple and lightweight indoor positioning design scheme proposed in this article is not only easy to popularize, but also provides new ideas for subsequent scientific research in the field of indoor positioning.

## 1. Introduction

In recent years, location-based service (LBS) has become the basic service of people’s daily work and life [1]. Global Navigation Satellite System (GNSS) plays an important role in LBS, which can provide stable and reliable location service in outdoor environment [2]. However, due to the serious signal occlusion in indoor environment, the availability of navigation satellite signal cannot be guaranteed. As the “Last Kilometer” in the field of positioning and navigation, it is of great significance to establish an accurate and reliable indoor positioning system to meet the public’s indoor positioning needs.

Generally, there are two main ways to achieve indoor positioning on a smartphone. The first way is a positioning method based on an external signal source, and the second way is a positioning method using a self-contained sensor. Specifically, in the first category, mainstream positioning methods include Wi-Fi fingerprint matching, that is, establishing a mapping relationship between position information that is difficult to directly measure and signal characteristics that are easy to obtain. In real-time positioning, positioning is achieved by matching the measurement signal with the fingerprint database [3,4]. The indoor positioning technology based on ultrasonic waves uses the sound generator installed in the indoor environment to emit sound signals, and uses the reflective distance measurement method to achieve positioning [5]. The indoor positioning technology based on the LED light source usually uses the quantitative relationship between the light intensity and the distance received by the receiving end to achieve indoor positioning [6]. In the positioning technology based on Bluetooth low energy, the proximity method and the fingerprint method are usually used for positioning. It has the advantages of low transmission power, high performance, and easy deployment, and has become a widely used positioning technology. The positioning accuracy is generally 3–5 m [7,8]. Pseudolite indoor positioning technology is a mainstream solution to seamless indoor and outdoor positioning and navigation. It mainly uses the carrier phase of pseudolite base stations deployed in the environment for high-precision ranging. The positioning accuracy is high and the deployment cost is relatively expensive [9]. The second type of positioning method mainly uses self-contained sensors on the smartphone to achieve positioning. With the development of micro-electromechanical systems (MEMS) technology, low-cost inertial measurement units have been widely used in most smartphone, such as accelerometers, gyroscopes and magnetometer, etc. [10], due to their small size, light weight, and low power consumption. Pedestrian dead reckoning (PDR) is a positioning technology that uses inertial sensor data to calculate the position of a pedestrian. Compared with positioning technology based on wireless signals and vision sensors, PDR can give an accurate position in a short time, the update of pedestrian position is faster, and the power consumption is lower. Furthermore, PDR systems are simpler and more autonomous because no additional infrastructure assistance is required. There are extensive researches on pedestrian dead-reckoning and its applications [11,12,13], the typical PDR algorithm mainly includes three parts: step detection, stride length estimation, and heading estimation. In the step detection process, acceleration data is usually used to analyze the number of steps walked, such as peak detection, zero crossing and flat zone detection [14]. In stride length estimation, commonly used methods include linear or non-linear estimation [15]. For heading estimation, researchers usually use accelerometers, magnetometers, and gyroscopes to determine the direction of pedestrians [16]. Using the above three steps and the initial position, continuous position prediction can be achieved.

However, the existing technology still has some limitations. Due to the limited accuracy of cheap MEMS sensors, a large cumulative error will occur after a long-term positioning solution, making the positioning results inaccurate. This also causes a single PDR method to not meet the needs of long-term indoor positioning. Many improvement methods have also been proposed for the problem of cumulative errors, it is hoped that the algorithm will minimize the impact of the sensor’s own accuracy. For example, the low-pass filtering method is used to reduce the random noise of the data [17]. In reference [18], the author proposed three error models for stride length estimation, which are the Gaussian model, constant random model, and Gaussian Markov model. Some scholars also established the fourth error model of stride length estimation on the basis of predecessors, and discussed the systematic error and random error of Taylor expansion [19]. In order to reduce the drift error of inertial devices in indoor environment, researchers proposed a scheme based on acceleration and gyroscope output to detect pedestrian attitude [20]. In addition, the scheme can also correct the heading of walking in real time and suppress the accumulated error of inertial position to a certain extent. According to the kinematic law, the author of reference [21] proposed a particle filter based on the adaptive weighted update strategy of hidden Markov model, which improved the accuracy of pedestrian navigation system, and carried out the corresponding simulation experiments. In paper [22], the author proposed a complementary filter and a gait detector to correct the cumulative error of pedestrian positioning. The error of direction estimation is mainly due to the magnetic field interference and gyro drift. In paper [23], the predictable errors of magnetic field, including hard iron and soft iron effects, magnetic declination, tilt and misalignment, are derived in detail, and a unified directional error model is obtained.

Although many algorithms have been used to correct the errors from the sensor itself, it is difficult to maintain stable and reliable positioning just by optimizing the sensor itself. Therefore, some scholars have thought of using the strategy of integration with the external positioning mode to calibrate the accumulated error. For example, in the joint positioning system based on MEMS inertial equipment and fingerprint, some researchers proposed a cascade filtering algorithm to improve the accuracy of indoor positioning [24]. Some scholars used the Sage–Husa adaptive Kalman filter to improve the accuracy and stability requirements of integrated positioning and navigation systems [25]. In reference [26], the author studied the fusion indoor positioning system using Bluetooth low energy and PDR, using beacons to fuse at the result level. In paper [27], a method of merging Wi-Fi and PDR to achieve positioning using the maximum likelihood method is proposed, and the advantages of PDR are used to eliminate the disadvantage of Wi-Fi. However, the stability of the positioning system is poor due to the randomness of the Wi-Fi signal.

Recently, due to its strong nonlinear fitting ability and deeper feature extraction ability, deep learning has become a research hotspot in various industries. Similarly, many scholars have introduced deep neural network models into the field of indoor positioning to process data from inertial devices and various signal sources in indoor environments. For instance, in reference [28], the author proposed an accurate estimation method of walking speed using deep learning for smartphone-based PDR. Some researchers applied a method to learn the speed of pedestrians by using segmented signal frames and mixed multi-scale convolution and recurrent neural network models, and estimated the travel distance by calculating the speed and moving time [29]. In the paper [30], the author used the deep learning method to process the pictures taken by the smartphone camera, then identifies the user’s location, and uses the particle filter algorithm constrained by the scene information to determine the final location. In reference [31], the author used machine learning method to analyze the self-contained MEMS sensor data in intelligent wearable devices, and then identified the activity characteristics of the elderly in the indoor environment, and finally constructed a set of intelligent home service system. In reference [32], the author used a stack Auto-encoder network to identify the characteristics of Internet of things data in an indoor environment, and to build a fingerprint database for online location. In reference [33], the author proposed a location method based on the fusion of Geomagnetism and MEMS of smart phone, and then identified the scene by constructing convolutional neural network to improve the location accuracy.

The above-mentioned indoor learning positioning strategy based on deep learning can basically achieve satisfactory positioning results. Generally, deep learning can play a key role in solving nonlinear problems. However, there are still some problems. For example, many deep neural network models are offline processing methods. When applied to smartphones, they will generate large power consumption, such as processing photos taken by cameras. Therefore, finding a lightweight network model and a reasonable application model have become the key issues. This paper mainly discusses how to use deep learning methods to process sensor data to achieve the purpose of calibrating the cumulative error of PDR, and finally build a lightweight indoor positioning system that is easy to promote and apply. The main contributions of our work are as follows:

1. In this paper, after reviewing the existing improved methods of PDR, we deeply study the methods of step number detection, step size estimation, and direction estimation. And we use the MEMS sensor on the smartphone to implement a continuous positioning system based on the PDR algorithm. Users can use this system to perform continuous positioning in an indoor environment, and the positioning results will be displayed on the smartphone screen in real time.
2. Aiming at the problem that the PDR algorithm will generate cumulative errors due to long-term positioning, a method for calibrating the cumulative errors by using indoor natural environment information is proposed. We have designed a new sparse Stacked Denoising Auto-Encoder (SDAE) model to process MEMS sensor data for the purpose of detecting turning points in indoor areas. In the algorithm description, we introduced in detail how the turning point region is defined and how the data set is constructed. At the same time, we elaborate on the structure and training process of the model. Finally, we compared the performance of the proposed model with commonly used machine learning classification algorithms through a large number of experiments. In the implementation of the positioning system, we propose a method of calibrating the cumulative error using particle filtering, and verify the effectiveness of the method through experiments.
3. In this article, we propose an innovative application method that saves a trained deep learning network as a KB-level model and stores it in the root directory of a smartphone. This model can respond quickly in real-time positioning and meet the real-time requirements of indoor positioning. In large-scale space and small-scale space indoor environments, we have verified through a large number of experiments that the proposed positioning system has significant performance improvements in positioning accuracy and stability. And we verified the adaptability of the model on different configurations of smartphones. We hope that the ideas in this paper provide new ideas for indoor positioning solutions based on smartphones.

The remainder of this paper is organized as follows: Section 2 introduces the structure of the positioning system, analyzes the implementation principle of the system and related preparations. Section 3 introduces the turning point detection algorithm based on deep learning network, and compares it with various commonly used machine learning methods to verify the classification performance of the proposed model. Then, in Section 4, the positioning performance of the proposed system is verified by a large number of experiments. In Section 5, we summarize the paper and provide a discussion about the benefits and limitations of this approach, future work to be done, and some final conclusions.

## 2. Preliminaries and Overview of System

In this section, the basic principles of PDR algorithm are introduced first. Then it describes in detail the implementation methods of the three component modules (Step Detection, Stride Length Estimation, and Heading Determination) of the PDR algorithm, including step number detection, step length estimation, and direction estimation, and verifies each module using the measured data. Finally, the system framework and implementation details presented in this paper are introduced.

### 2.1. Pedestrian Dead Reckoning

**Figure 1:**
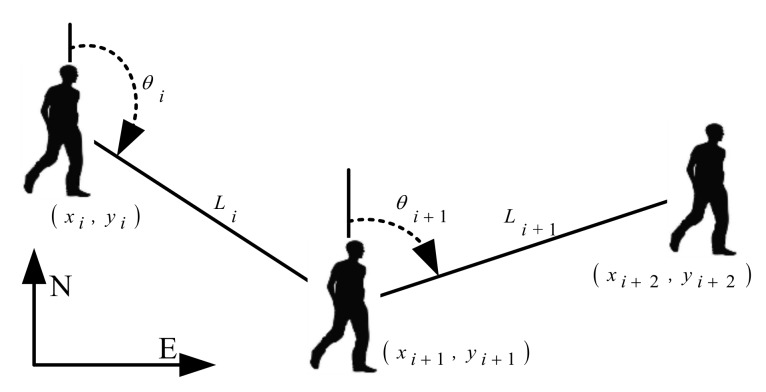
Schematic of pedestrian dead reckoning (PDR).

The PDR algorithm is based on the gait characteristics of pedestrian walking and uses a low-cost self-contained sensor to calculate the relative position of the pedestrian. Figure 1 is a schematic diagram of the PDR algorithm. Given the initial position, the position of the pedestrian can be updated by Equation (1).

(1) $$\left\{\begin{array}{l} x_{i+1}=x_{i}+L_{i}\cdot \sin(\theta_{i})\\ y_{i+1}=y_{i}+L_{i}\cdot \cos(\theta_{i}) \end{array}\right.$$

where $x_{i}$ is the east–west coordinate of the pedestrian in the northeast coordinate system at step i; $y_{i}$ is the north-south coordinate of the pedestrian in the northeast coordinate system at step i. L is the step size, and θ is the direction of travel.

#### 2.1.1. Step Detection

During the normal walking process, pedestrians’ own acceleration changes periodically. Researchers often use the acceleration sensor of smartphones to detect the number of pedestrian steps. Generally, the data acquired by the accelerometer include acceleration in three directions $\left(a_{x},a_{y},a_{z}\right)$. In this article, Equation (2) is used to calculate the modulus of the three-axis acceleration to detect the number of steps.

(2) $$a_{mer}=\sqrt{a_{x}^{2}+a_{y}^{2}+a_{z}^{2}}$$

**Figure 2:**
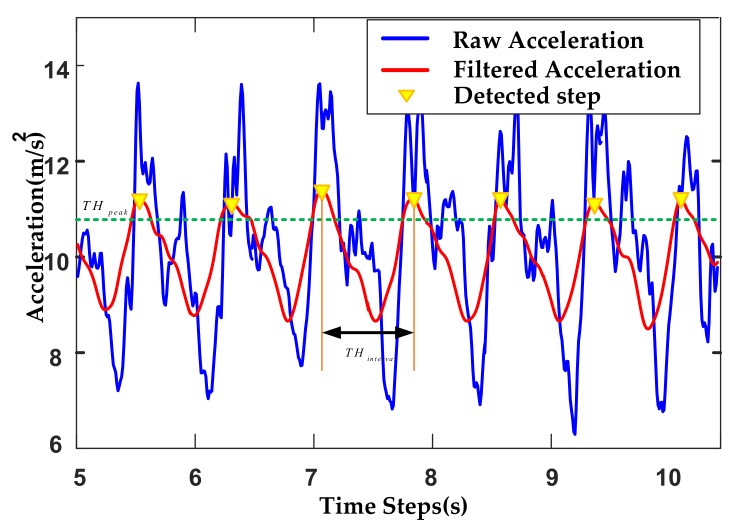
The raw and filtered accelerations.

Through the analysis of sensor signals in different motion modes, we find that most of the signal frequencies are lower than 10 Hz, so we use a low-pass filter with a cut-off frequency of 10 Hz to suppress high-frequency noise. The signal after average filter and low-pass filter is shown in Figure 2. Compared with the original signal, the filtered signal noise is smaller and smoother, so it can reflect the characteristics of pedestrian movement more clearly. Based on the filtered acceleration data, two constraints are used to determine each step.

- Peak value must be greater than the given threshold $TH_{peak}$;
- The interval between the peak value and the adjacent valley value must be greater than the given threshold value $TH_{interval}$.

#### 2.1.2. Stride Length Estimation

The stride length of pedestrians is random, so it cannot be set to a fixed value in the PDR positioning algorithm. For different individuals, stride length will be affected by height, gender, and walking speed. However, for the same pedestrian, the stride length is mainly related to the pedestrian’s stride frequency. Researchers have proposed many estimation models, most of which are calculated using accelerometer data, including linear models, nonlinear models, and artificial neural network models. This paper uses the nonlinear to estimate the pedestrian stride length, which is expressed as:

(3) $$L=K_{1}a_{avg}T_{step}+K_{2}\sqrt[{4}]{a_{\max}-a_{\min}}.$$

Among them, $K_{1}$ and $K_{2}$ are the coefficients of the model, $a_{avg}$ is the average value of the acceleration measured at this time epoch, and the unit is $m/s^{2}$. $T_{step}$ is the walking time of each stride, and $a_{\max}$ and $a_{\min}$ are the maximum and minimum accelerations measured this tim

#### 2.1.3. Heading Determination

In the experimental environment, the walking direction of pedestrians can be estimated effectively. However, in the real indoor environment, due to the influence of hard iron and soft iron, the direction data is easy to be interfered, and then the location results of PDR will be greatly error. Therefore, some researchers combine the information of accelerometer, gyroscope, and magnetometer to obtain relatively reliable direction estimation. In this paper, a Madgwick filter [34] is used as an absolute direction capture method because of its good performance, low computational complexity, and real-time processing capability on non-high-precision sensors. Since the attitude is relative, we take the horizontally stationary north direction as the initial attitude. After the pedestrian moves, the quaternion ${}_{S}^{E}q={\left[q_{0}\;q_{1}\;q_{2}\;q_{3}\right]}^{T}$ can be used to indicate the relative attitude to the initial attitude. The essence of Madgwick filter used to fuse sensor data is to weigh the attitude calculated by gyroscope and the attitude calculated by accelerometer and magnetometer at a specific time. Their weight coefficients are determined by the proportion of their respective errors and total errors. The smaller the error ratio is, the larger the weighting coefficient is. It is worth mentioning that the sampling frequency of the gyroscope is usually much faster than the accelerometer and magnetometer. In actual applications, the sampling frequency of the sensor is often set to the minimum of the three to ensure the data synchronization of the sensor. The filter output is an estimated quaternion (four-dimensional vector) based on actual sensor measurements. As shown in Equation (4).

(4) $$\begin{array}{l} Yaw=\arctan2\left(\frac{2\left(q_{1}q_{2}+q_{0}q_{3}\right)}{q_{0}^{2}+q_{1}^{2}-q_{2}^{2}-q_{3}^{2}}\right)\\ Pitch=-\arcsin\left(2\left(q_{1}q_{3}-q_{0}q_{2}\right)\right)\\ Roll=\arctan2\left(\frac{2\left(q_{0}q_{1}+q_{2}q_{3}\right)}{q_{0}^{2}-q_{1}^{2}-q_{2}^{2}+q_{3}^{2}}\right) \end{array}$$

**Figure 3:**
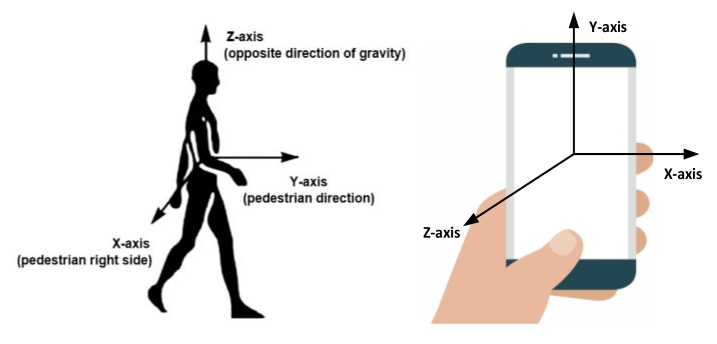
Schematic diagram of heading definition.

Here, the quaternion vector is used to calculate the heading angle, pitch angle, and roll angle. It should be pointed out that the algorithm proposed in this paper is based on a premise, that is, the *Y*-axis of the smartphone approximately points to the travelling direction Yaw of the pedestrian, as shown in Figure 3.

**Figure 4:**
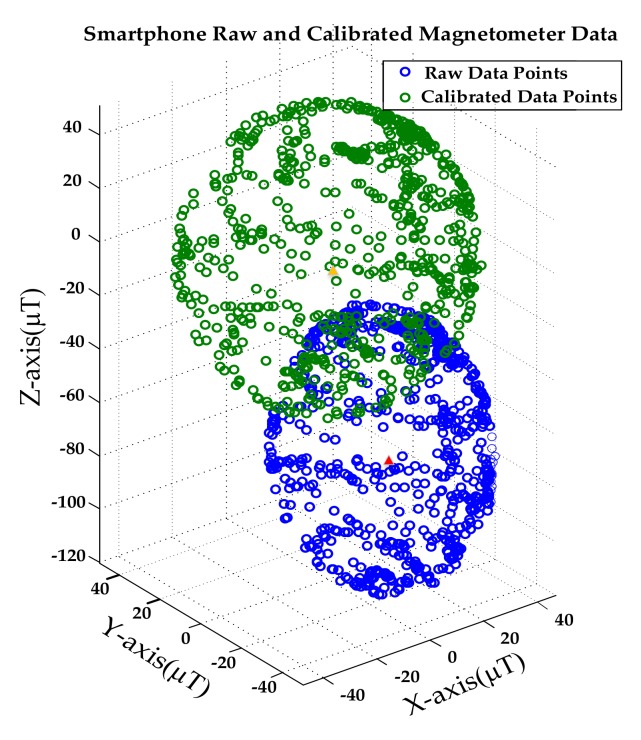
Blue dots are un-calibrated raw data and green dots are calibrated data.

However, before obtaining the absolute direction of the user, due to the uncertainty of the user’s initial state, the interference of the soft iron or hard iron in the surrounding environment will cause the initial value of the magnetometer not to be horizontally stationary to the north. In this case, there is an error in the initial attitude determined by the accelerometer and the magnetometer, so some methods are often used to calibrate the error of the magnetometer. In this work, we chose the ellipsoid calibration algorithm because it makes a good trade-off between calibration accuracy and data. The algorithm collects measurement results from all possible sensor positions in space to correct the deviation, and distributes the corrected values on a standardized sphere as shown in Figure 4. While the user is walking, the magnetometer will still be subject to various disturbances from the surrounding environment, so we need to use the optimal attitude estimation value at the last moment to calibrate the value of the magnetometer in real time to ensure the accuracy of attitude estimation.

### 2.2. Overview of System

**Figure 5:**
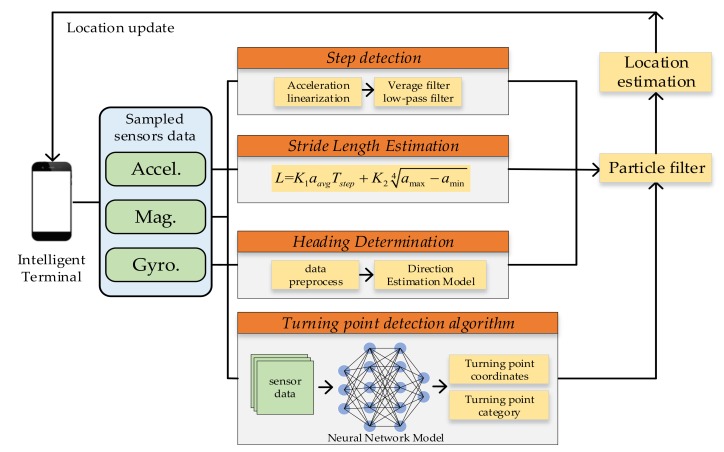
Positioning system architecture diagram.

In the indoor positioning system proposed in this paper, we use the MEMS sensor on the smartphone to implement the PDR algorithm, which includes three modules: step detection, stride length estimation, and heading estimation. Aiming at the problem of cumulative error of the PDR algorithm, we designed a turning point region detection algorithm based on a deep neural network model. In the offline phase, multiple testers use smartphones to collect sensor data from different turning areas, and use the preprocessed data to build a data set. After training, the trained model is stored in the smartphone. In the real-time positioning phase, the turning points identified by the turning point detection model are fused using a particle filter algorithm to calibrate the cumulative error of the PDR. The framework of the system is shown in Figure 5.

## 3. Proposed Method and Implementation Details

Due to the influence of the surrounding environment interference and the measurement error of the sensor, the long-term positioning of the PDR-based positioning system will produce a large cumulative error. Low-precision MEMS sensors in smartphones alone cannot achieve high-precision positioning for a long time. As we all know, when the indoor environment is completed, the indoor walking route will not change greatly, such as the corners of the corridor and the position of the door of the room can be used as environmental features. Therefore, after adding labels to different turning points, combined with the prior information of the map, a set of feature libraries unique to the indoor environment can be constructed. Therefore, in the first part of this chapter, we will introduce how to plan and detect the turning point area in an indoor environment. At the same time, the construction method and implementation details of the turning point detection model are introduced in detail. In the second part, we explain how to use particle filter algorithm to fuse the detection model and MEMS sensor data, and finally realize a high precision indoor positioning system.

### 3.1. Turning Points Detection Algorithm Based on Machine Learning

In the indoor environment after the completion, due to the block of the building structure, many turning areas are formed in the indoor area. Therefore, we can collect sensor data in the feature area and build a turning fingerprint database to detect the turning point.

#### 3.1.1. Building Training datasets

**Figure 6:**
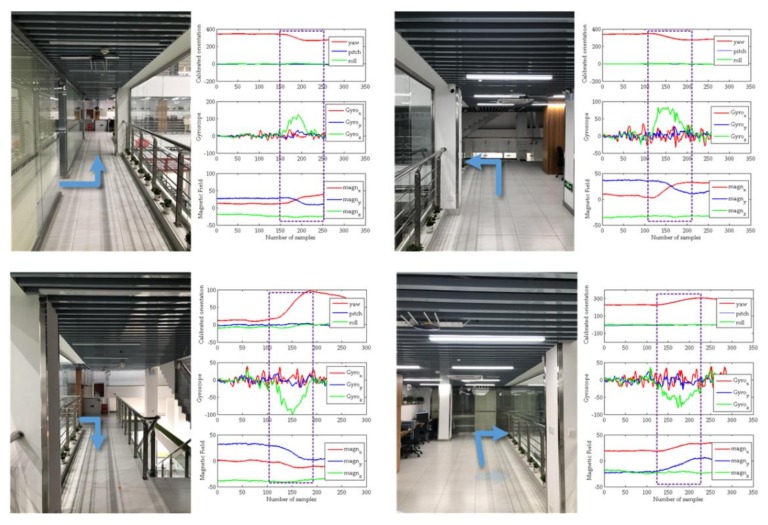
Characteristic data of different turning points.

In the offline process, we define 8 direction categories of “Northern ↔ East”, “East ↔ South”, “Southern ↔ West” and “Western ↔ North”. The orientation here is the relative orientation we defined. Considering the actual problem, since the turning angle may not be strictly right angle, we use deep learning algorithm to extract the changing characteristics of the sensor data when turning. For example, from north to east, the characteristic change trend in the set sliding window can be regarded as the pedestrian turning from the north to the east. At the same time, when constructing the data set, we consider the data of the geomagnetic field as a feature to increase the discrimination of the turning point category. In the sub-picture of Figure 6, the left picture shows a schematic diagram of the turning area in the test scene, and the right picture shows the data changes of the mobile phone sensor, where the data includes orientation data, gyroscope data and geomagnetic field data.

In practical applications, considering the specific indoor environment structure, we can also take a variety of special cases such as continuous left turn, continuous right turn, first left to right, first right to left as new categories, and add them to the training data set.

#### 3.1.2. Detection Model Based on Deep Learning

Smartphone sensor data can not only provide location predictions, but also be able to sense user behavior information in an indoor environment. Research in this area is often called Human Activity Recognition (HAR) technology. This paper considers the problem of turning points detection as a time series multi-classification problem, and more representative features can produce better classification results. After constructing a suitable training data set, a deep learning model is used to establish the mapping relationship between the sensor data features and the regions of different turning points.

**Figure 7:**
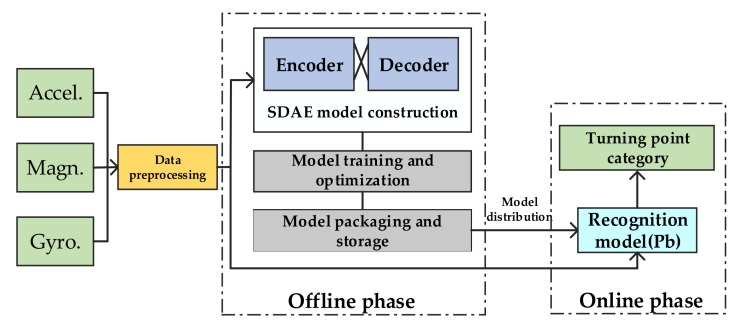
Infrastructure of the turning point detection model.

Considering the high time complexity of using the traditional methods in the field of signal processing to identify the Turning point directly from the original sensor data, the features extracted manually are limited by the field of human knowledge, so this paper designs a lightweight deep learning network model to extract the deep features in the data to identify the turning point, which improves the recognition efficiency and accuracy. In the model selection of turning point detection, we mainly consider two factors. In the first aspect, currently commonly used machine learning algorithms such as Support Vector Machine (SVM), shallow neural networks, etc. can complete the task of signal feature extraction. However, we know that in the case of machine learning algorithms, when the hidden layer is reduced by one layer, the number of hidden units required to maintain the same data representation capability increases exponentially. Therefore, similar to the above-mentioned several shallow algorithms, it is difficult to fully learn the data features, which leads to the performance degradation of unsupervised and incremental tasks. The traditional pattern method is limited in terms of classification accuracy and model generalization performance. In the second aspect, sensor data is deterministic continuous data. Compared with probabilistic models (such as deep belief network (DBN)), numerical models are more suitable for solving this problem, so this paper chooses SDAE. The model can learn more representative deep features from the noisy data, and can effectively deal with over-fitting problems in deep learning network training, improve the robustness of the detection algorithm, and then improve classification accuracy. The basic framework of the turning point recognition model is shown in Figure 7.

Generally, after the Autoencoder training is completed, only the encoder is used to complete the next work. We connect the Softmax layer before the output layer to complete the multi-classification task. The specific construction method and training method of the model are as follows: DAE and traditional AE have the same structure, but noise is added according to certain principles during sample input. The function implemented by DAE is to learn the original data with noise added, and learn more representative features from the noise data. SDAE is a stack of multiple DAEs, which also includes input layer, hidden layer, and output layer. The pre-processed data is abstracted and extracted by multiple hidden layers, and the output of each hidden layer is a feature representation. For deep neural networks, if the traditional random initialization parameter method and gradient descent training are used, the weight parameters of some hidden layers will be insufficiently trained, and the optimal parameters of the network cannot be obtained. Therefore, we use the layer-by-layer training method, which includes network pre-training, network expansion, and network fine-tuning. In network pre-training, each hidden layer is composed of a DAE, and each layer is trained independently to find local optimal parameters.

We add noise with a certain probability distribution to the input of the autoencoder network, so that the network learns the ability to remove this noise, makes its learned features more robust, and improves the model’s generalization ability to the input data. Compared with the traditional DAE method of removing some data according to a certain rule (binomial distribution), the noise reduction method proposed in this paper is more suitable for analyzing low-precision sensor data. In network design, if the number of nodes in the hidden layer is less than the number of nodes in the input layer, a reduced-dimensional representation of the original data will be obtained, that is, the internal correlation between the data. However, this will lead to the inability to learn the sensor data features in depth. Therefore, this article chooses to set the number of hidden layer nodes greater than the input layer nodes, and increases the sparsity limit, so that some hidden units are activated. In this way, not only the representative features can be deeply mined, but also too many data features will not be lost, and the network will not learn “identity relations”. The output cost function of the model can be expressed as Equation (5).

(5) $$J_{s}\left(w,b\right)=J\left(w,b\right)+\beta \sum_{j=1}^{h}KL\left(\rho \left\|{\bar{\rho }}_{j}\right.\right)+\alpha \Omega \left(w\right)$$

Here, w and b represent the training parameters of the model, the first term $J\left(w,b\right)$ is the cost function of the network model, the second term is the sparse constraint term, and the third term $\alpha \Omega \left(w\right)$ is the penalty term for the weight parameter, where α is $\alpha =\left[0,\infty \right)$. Specifically, the first term can be written in the form of Equation (6), where $L\left(x,g\left(f\left(\tilde{x}\right)\right)\right)$ represents the loss function of the input data of the autoencoder as shown in Equation (7), here $\tilde{x}$ is the input data for adding noise according to the rules. Functions $f\left(\cdot \right)$ and $g\left(\cdot \right)$ are non-linear activation functions used to form encoders and decoders. We choose the Sigmoid function.

(6) $$J\left(w,b\right)=\frac{1}{2m}\sum_{i=1}^{m}L\left(x^{\left(i\right)},g\left(f\left({\tilde{x}}^{\left(i\right)}\right)\right)\right)$$

(7) $$L\left(x,g\left(f\left(\tilde{x}\right)\right)\right)={\left\|x-g\left(f\left(\tilde{x}\right)\right)\right\|}^{2}$$

In the second term of Equation (5), β is the weight of the sparse penalty factor, h is the number of hidden layer units, and ρ is the sparseness parameter. According to the introduction of UFLDL [35], the activation degree of hidden unit j is $a_{j}^{\left(2\right)}\left(x\right)$ on the given input data x. ${\bar{\rho }}_{j}$ is the average activation degree of the jth hidden unit over the entire training data set, that is ${\bar{\rho }}_{j}=\frac{1}{m}\sum_{i=1}^{m}\left[a_{j}^{\left(2\right)}\left(x^{\left(i\right)}\right)\right]$, and m is the number of input data. $KL\left(\rho \left\|{\bar{\rho }}_{j}\right.\right)$ is the relative entropy (KL divergence) [36], which represents the relative entropy between two Bernoulli random variables with ρ as the mean and ${\bar{\rho }}_{j}$ as the mean. KL is a standard method for measuring the difference between two distributions, which is Equation (8). When the difference is larger, the penalty is stronger, ensuring that only a small number of hidden units are active.

(8) $$KL\left(\rho \left\|{\bar{\rho }}_{j}\right.\right)=\rho \log\frac{\rho }{{\bar{\rho }}_{j}}+\left(1-\rho \right)\log\frac{1-\rho }{1-{\bar{\rho }}_{j}}$$

The third term in Equation (5) can be regarded as a regular term. Its purpose is to prevent the objective function from over-fitting. Usually, $\Omega \left(w\right)={\left\|w\right\|}_{2}$ is used for L2 regularization.

Through the above formula, we can calculate the cost function of the network. If it is less than the set threshold, that is $J_{s}\left(w,b\right)<\varepsilon$, the training is over, and the current parameters are the output parameters of the final model. In order to realize the multi-classification problem, we use the Softmax layer as the output layer, and its input layer is the last hidden layer of the autoencoder. According to the hypothesis function $h_{w}\left(x\right)=\frac{1}{1+\exp\left(-w^{T}x\right)}$, for each input data x, calculate the probability $p\left(y=j\left|x\right.\right)$ of the category j, where w is the model weight parameter. The error cost function is shown in Equation (9).

(9) $$J\left(w\right)=-\frac{1}{m}\left[\sum_{i=1}^{m}\sum_{j=0}^{n}I\left\{y^{\left(i\right)}=l\right\}\log\frac{e^{w_{l}^{T}x\left(i\right)}}{\sum_{l=0}^{n}e^{w_{l}^{T}x\left(i\right)}}\right]+\alpha \Omega \left(w\right)$$

**Figure 8:**
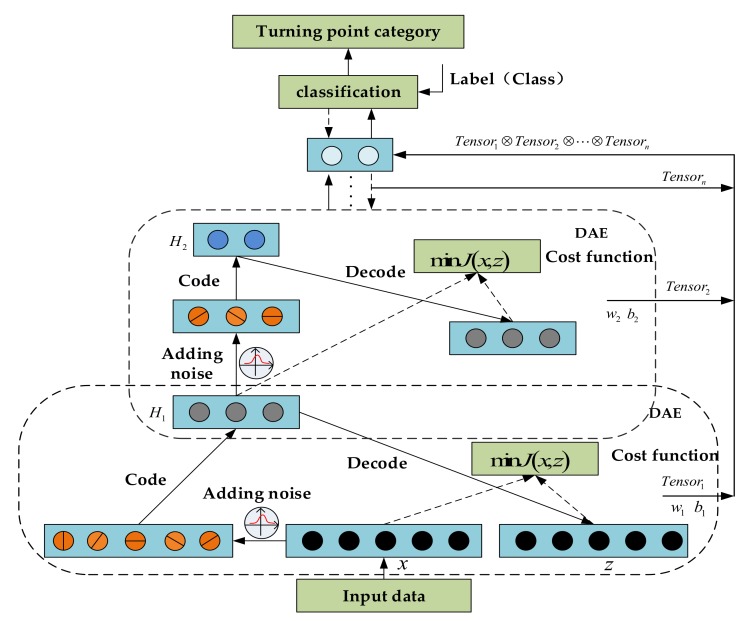
Turning point detection model based on Stacked Denoising Auto-Encoder (SDAE) network.

Among them, w is the weight parameter of the classifier, m is the number of input samples, n is the number of categories, $I\left\{\cdot \right\}$ is the truth function, and $\alpha \Omega \left(w\right)$ is the regular term. At this time, the autoencoder and classifier are stacked to perform network expansion and fine-tuning. At the same time, the global parameters of the network are adjusted by the gradient descent method, and then the global optimal solution is obtained. The structure of the turning point recognition model is shown in Figure 8:

The input layer of the network is used as the input of the first DAE layer, and the input after adding random noise is used as the output of the first DAE layer. The features of the first layer are obtained by learning from the encoder. Similarly, the output of the first layer is used as the input of the second DAE layer, and the second layer features are obtained, and so on. Compared with SVM, the Restricted Boltzmann Machine (RBM) [37], and traditional autoencoder, the stacked form of SDAE, its powerful learning ability, and low error rate are other reasons for choosing this model. Each layer works independently, and features Ts are extracted separately. Among them, Ts is a finite-dimensional linear space. Each layer is characterized by a tensor. Finally, the tensor of each layer is fused as shown in Equation (10):

(10) $$Ts=Ts_{1}⊗Ts_{2}⊗\cdots ⊗Ts_{k}$$

Any element of it can be represented as $\gamma_{1}⊗\gamma_{2}⊗\cdot ⊗\gamma_{k},\;\;\gamma_{i}\in Ts_{i},\;\;i=1,2,\cdot ,k$. Among them, $Ts_{k}$ is the output feature of the DAE at the k th layer. The final Ts is used as the input feature of the basic network classification model to obtain the largest linear subspace in the bilinear space, which improves the robustness of the network model.

**Figure 9:**
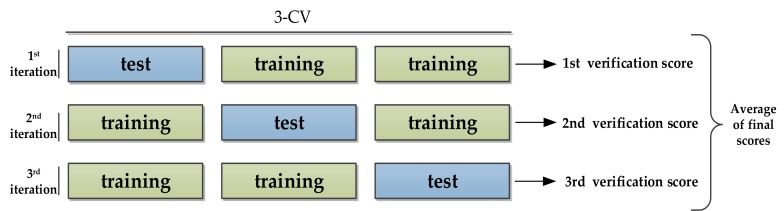
Three-fold cross validation.

After the network model is constructed, the network parameters are initialized, and each DAE network is pre-trained with unlabeled data. Then use the output features of the encoder to build a classifier, also initialize the parameters, use the labeled data for fine-tuning, and finally obtain the turning point classification model. In order to adjust network parameters during training, we use grid search to optimize network parameters. When evaluating the model, due to the limited number of data sets, we use K-fold cross validation to instantiate K identical models, train each model on K-1 partitions, and use the remaining partition for evaluation. The model’s validation score is equal to the average of the K validation scores. The 3-fold cross validation diagram is shown in Figure 9.

In the real-time positioning phase, the positioning system detects turning points through the turning point detection algorithm. After the turning point category is identified, the turning point coordinates are assigned to particle filtering for fusion positioning.

### 3.2. Fusion Calibration Algorithms Based on Particle Filtering

In this section, we introduce the implementation details of calibrating PDR cumulative error using particle filter fusion turning point coordinates. Generally, on many positioning and navigation problems, researchers choose different filtering methods according to different applications. We introduce particle filtering as a method of target tracking and result smoothing based on the user’s motion characteristics in indoor positioning. The particle filter method actually uses Monte Carlo method to simulate a large number of particles, and each particle has a state and weight. The state distribution and weight of all these particles simulate the probability distribution of the user’s position (or state). We can directly take the weighted average of the states of all particles to get the estimated position of the pedestrian. In the prediction process, set some rules (state transition) for the particles based on experience, such as moving each particle at a uniform speed, and resetting the position after encountering the calibrated area. At the same time, some randomness is added to the particles, so that various states of motion can be simulated. Then, the probability distribution brought by the position of the turning point after calibration is used to update the weight of each particle, and the particles that more closely match the observation result should be given greater weight. The state transfer equation of particles is shown in Equation (11) and the observation equation is shown in Equation (12):

(11) $$x_{t+1}=Ax_{t}+B_{u}u_{t}+w_{t}$$

(12) $$y_{t}=H_{t}x_{t}+e_{t}$$

Here $x_{t}$ represents the state vector (particle position) of the particle, $u_{t}$ represents the measured value, which may not be accurate, sometimes it may be 0, so there is a Gaussian error value $w_{t}$. From the perspective of probability distribution, the probability of the current location near the prediction point is larger, and the farther away the probability is smaller. $y_{t}$ is the observed value. In target tracking, $H_{t}$ represents the linear Gaussian relationship between $y_{t}$ and $x_{t}$, and $e_{t}$ is the measurement error. Both the prediction process and the observation process have a probability distribution of the target location. We choose a location with the largest joint probability as the estimation value. In this paper, A and H are unit matrices, $y_{t}$ is the coordinate value of inflexion point, and the state of each particle includes the particle position and probability distribution $P\left(x_{t}\left|z_{t}^{i}\right.\right)$, and its expression is shown in Equation (13).

(13) $$P\left(x_{t}\left|z_{t}^{i}\right.\right)=\frac{1}{\sqrt{2\pi \sigma }}\exp\left[-\frac{{\left(X_{t}-X_{z_{t}^{i}}\right)}^{2}}{2\cdot \sigma^{2}}\right]$$

Here $X_{t}$ is the observation position, that is, the coordinates of the turning point, $X_{z_{t}^{i}}$ is the position of the ith particle at time t, and σ is the measurement variance. Therefore, the specific implementation is as follows:

(1) Initialization. The randomly generated particle set includes the number of particles, the range of movement, the speed and direction of the movement, and set thresholds. Since we were unable to determine the state of the particles at the beginning, we believe that the particles are uniformly distributed in the full state space.
(2) Prediction. The state of each particle is predicted according to the state transition equation.
(3) Weight update. After the turning point category is identified through the turning point detection model, the position coordinates of the turning point are used as the observation result to update the weight of the particles, and the particles that are closer to the observation position get a larger weight.
(4) Resample. Duplicate some high-weight particles while removing some low-weight particles.
(5) Position estimation. The current state of the user is estimated from the particles and weights.

The specific implementation is shown in Algorithm 1.
**Algorithm 1:** Fusion Process Based on Particle Filter**Input:** Particle range: range_x, range_y. The number of particles: particles.Set particle moving speed: V. Initialization weight: weights.Initialization positioning: $P_{0}\left(x_{0},y_{0}\right)$.Calibration area ε;Turning point g data sequence $\delta_{g}$;Position coordinates of the turning point g: $P_{g}\left(x_{g},y_{g}\right)$.**Output:** Tracking results using particle filter and turning point calibration: state.
//$f\left(\delta \right)$ is the turning point detection algorithm;
1: **Initialization:** sample a set of particles from the initial state distribution2: **while** a new motion measurement **do**3: Current location update by formula(1);4: **for** each particle **do**5: Prediction: predict particle state by state transition equation;6: **if** Current location ∈
ε and $f\left(\delta_{g}\right)=true$
**then****7:**  Weighting: update particle weights by $P_{g}\left(x_{g},y_{g}\right)$;8: **else**9: Weighting: update particle weights by predicted location;10: **end if**11: **end for**12: **if** 1/sum(square(weights)) <length(particles)/2 **then**13: Resample: generate new particles based on their weights(multinomial resample);14: Weighting: update particle weights15: **end if**16: **if**
${\left\|state-P_{0}\right\|}_{2}$> threshold **then**17: Eliminate the location result at this time(outliers)18: Update the Current location using state19: **end if**20: **end while**

## 4. Implementations and Evaluation

In this section, we experimentally test the performance of the proposed positioning system. First, in the offline phase, many testers collect sensor data and build data sets based on the actual indoor environment for training network models. Then, testers walked along the prescribed route with the smartphone and obtained real-time sensor data. The receiver operating characteristic (ROC) and AUC (Area under Curve) standards are used to compare the classification performance of the turning point detection algorithm proposed in this paper with other commonly used classification models. Finally, the positioning performance of the proposed positioning system is tested in the large-scale space environment and the small-scale space environment from the two aspects of average positioning accuracy and cumulative error distribution function. And in the experiments, we selected mobile phones with different configurations that are common in the market to verify the adaptability of deep learning models and positioning systems.

### 4.1. Turning Point Detection Experiment

During the data set construction phase, we selected 10 testers to hold the smart phone and keep the handheld mode in Figure 3 to walk in the experimental environment. Collecting data from mobile phone sensors during walking, including directional time series $Attitude\left[roll,\;pitch,\;yaw\right]$ and geomagnetic field time series $GeoM\left[GeoMag_{x},\;GeoMag_{y},\;GeoMag_{z}\right]$ in the sliding window, six feature vectors are used as the input of the network model, and eight different turning point categories are used as the output of the model. We experimentally set the time step of the sliding window T = 2 s, respectively with 50% overlap between consecutive windows, the size of the sliding window is 100 samples and the sensor sampling frequency $f_{s}$ = 50 Hz. Here, the data in each sliding window represents an input sample. Considering the timeliness of the model and the effect of feature extraction, we design the deep neural network model with three hidden layers, each corresponding to an autoencoder, and the number of hidden units is 256-256-128, the output layer is set to eight categories. Some hyperparameter settings of the network are obtained through trial and error, and are set as shown in Table 1.

**Figure 10:**
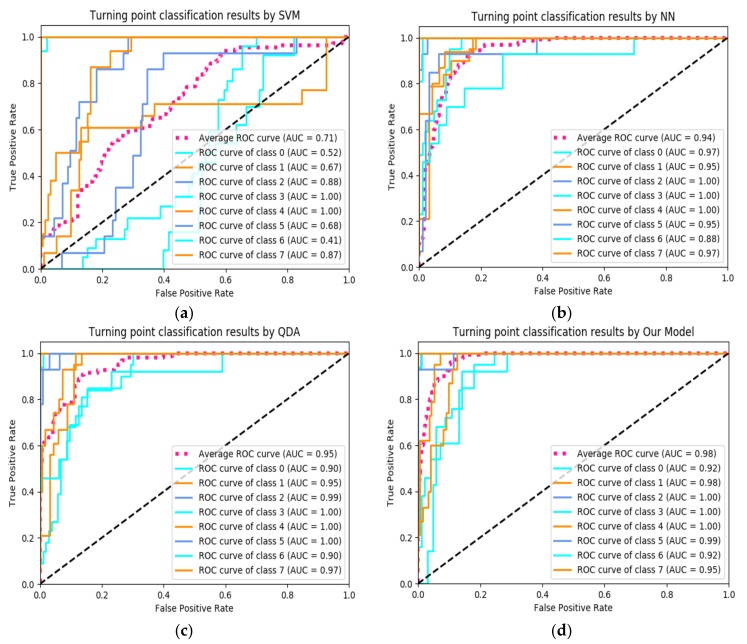
Performance comparison of common machine learning classifiers: (**a**) Classification Performance of Support Vector Machine (SVM); (**b**) Classification Performance of Neural Networks (NN); (**c**) Classification Performance of Quadratic Discriminant Analysis (QDA); and (**d**) Classification Performance of our model.

During the training phase, we used 3500 sets of data from seven testers as training data, and 1500 sets from the other three testers as test data. At the same time, we will binarize the output categories to facilitate the training of multi-class models. The label “1” indicates correct classification and the label “0” indicates classification errors. The trained turning point detection model is stored in the local memory of the smartphone. During the test phase, the tester held the smart phone in the test area. In real-time positioning, the detection model we designed will continue to detect the turning points through the real-time sensor time series in the sliding window. When the turning point area is detected, the next calibration operation is performed. In this article, we use four commonly used machine learning classification models in sklearn on the python environment including (Support Vector Machine, SVM), (Neural Networks, NN), and (Quadratic Discriminant Analysis, QDA) to compare the recognition performance of the proposed model. In SVM, we optimize the parameters by calculating the value of AUC. The penalty coefficient C is set to 3, the coefficient of the kernel function ‘gamma’ is set to the default value ‘auto’, and the degree is 3. In NN, we set the number of hidden units to 500, learning rate = 0.001, dropout = 0.5, and the optimizer set to ‘AdamOptimizer’. In QDA, the prior probability is ‘None’, the regularization parameter is 0, and the iterative convergence threshold is 0.0001. Generally, the ROC curve has a very good characteristic, that is, when the distribution of the positive and negative samples in the test set is transformed, the ROC curve can remain unchanged. In actual data sets, sample imbalances often occur, that is, the difference between the positive and negative samples is large, and the positive and negative samples in the test data may also change over time. So we choose the ROC curve to evaluate the quality of the multi-classification problem, use ROC-curve to calculate the distance between each sample and the decision boundary of each class, and then compare the performance of various multi-classification methods on the data set we build. The test results are shown in Figure 10.

In Figure 10a–c, and d the abscissa is (False Positive Rate, FPR), and the ordinate is (True Positive Rate, TPR). The larger the abscissa is, the more actual incorrect categories among the predicted correct categories. The larger the ordinate is, the more actual correct categories among the predicted correct categories. The ideal classification ROC curve is that the closer to the (0, 1) point, the better the classification effect. In order to show the quality of the classifier more intuitively, AUC (Area under Curve) is usually introduced to represent the area under the ROC curve, as shown in Equation (14). The value of AUC is between 0.1 and 1, and the larger the value, the better the performance of the classifier. Under Python3.0, the tensorflow1.5 deep learning framework and Scikit-learn0.19.1 are used to implement the network model. We got 98.2% test accuracy, it took 5760.2 s for training on CPU (Lenovo-idea-pad 700; Intel Core i5, 2.30 GHz; Mem 16 GB DDR3 1867 MHz), and the validity of our classification model in turning point detection is verified.

(14) $$AUC=\frac{\sum_{i\in positiveClass}rank_{i}-\frac{M\left(1+M\right)}{2}}{M\times N}$$

Among them, M is the number of positive samples and N is the number of negative samples. First, the scores are sorted from large to small (assuming that the larger the score, the greater the probability that this sample belongs to the positive category). $n=\left(M+N\right)$ is the total number of samples. The sample rank corresponding to the largest Score is n.

In human activity recognition problems, the size of the sliding window plays an important role. If the sliding window is set too small, the data in the window cannot guarantee a complete action. If the window sequence is too large, it will contain more than one action, which will reduce the recognition accuracy. Therefore, for the turning point recognition problem, in order to find a suitable window size, we have chosen the varying windows size of 0.5, 1, 1.5, 2, 2.5, 3, 3.5, and 4 s with 50% overlap. The experimental results are shown in Table 2.

According to the actual problem, the size of the window is closely related to the usage model. As can be seen from the above table, considering the real-time requirements of indoor positioning and the recognition accuracy of the model, it is more appropriate to set the time step of the sliding window to 2 s.

### 4.2. Localization Experiment

Above, we verified the effectiveness of the turning point classification model. In this section, we will perform positioning performance experiments in small-scale indoor areas and large-scale indoor areas. The test content includes the positioning accuracy of the positioning system and the adaptability in differently configured smart phones, and the test results are clearly given through data analysis.

**Figure 11:**
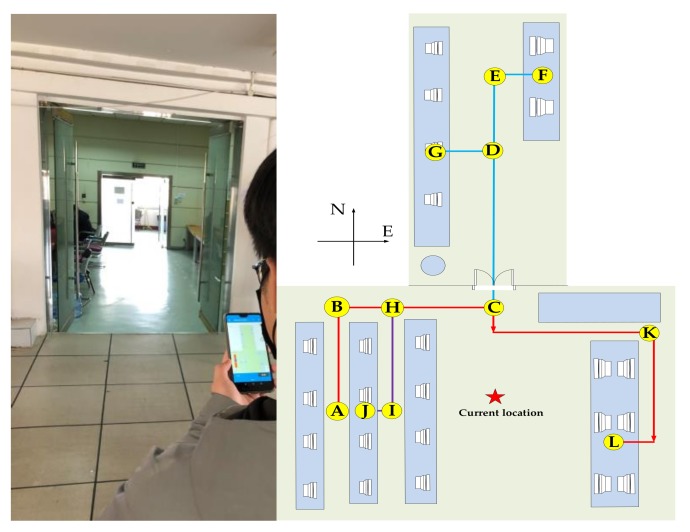
Small scale indoor test environment.

(1) Experiment 1: In a real office environment with an area of about 124 m^2^, we planned four different travel routes: Path 1: A→B→C→E→F, path length is 21 m, including three turning points; Path 2: A→B→C→D→G path length is 20 m, including three turning points; Path 3: A→B→H→I→J path length is 17 m, including three turning points; Path 4: A→B→C→K→L path length is 28 m, including five turning points as shown in Figure 11.

**Figure 12:**
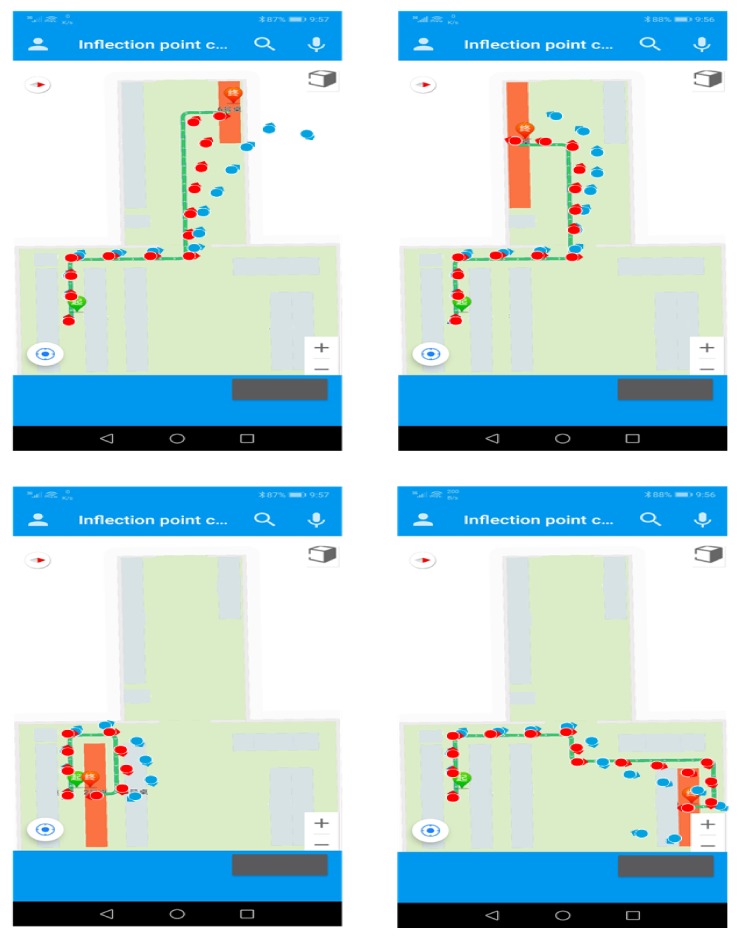
Positioning test results in a small-scale indoor environment.

Four volunteers were selected to hold a smartphone with the positioning application software to walk along the prescribed route in the test environment, and record the positioning results and walking trajectories. The experimental results are shown in Figure 12.

**Figure 13:**
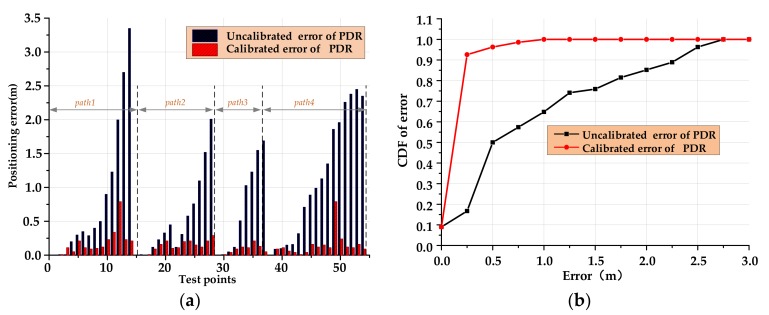
Statistical analysis of small-scale indoor environment: (**a**) linear distance error and (**b**) cumulative error distribution function.

In Figure 12, blue marks are the trajectories of the PDR without calibration, and red marks are the results after fusion calibration. It can be clearly seen that the accuracy of the positioning results after calibration is higher, and the overall travel trajectory is closer to the real trajectory. In order to better compare the positioning accuracy, we have calculated the positioning results as shown in Figure 13.

As can be seen from Figure 13, in the small-scale indoor space environment, the average positioning error of the original PDR algorithm is 0.83 m, the maximum positioning error is 3.3 m, the probability of the error is within 0.5 m is 50%, and the probability of the error is within 1 m is 65%. After the turning point information is fused by particle filtering, the average positioning error is 0.14 m, the maximum positioning error is 0.76 m, the probability of the error within 0.5 m is 96%, and the probability of the error within 1 m is 100%, which verifies the effectiveness of the proposed algorithm. Statistics of positioning errors are shown in Table 3.

**Figure 14:**
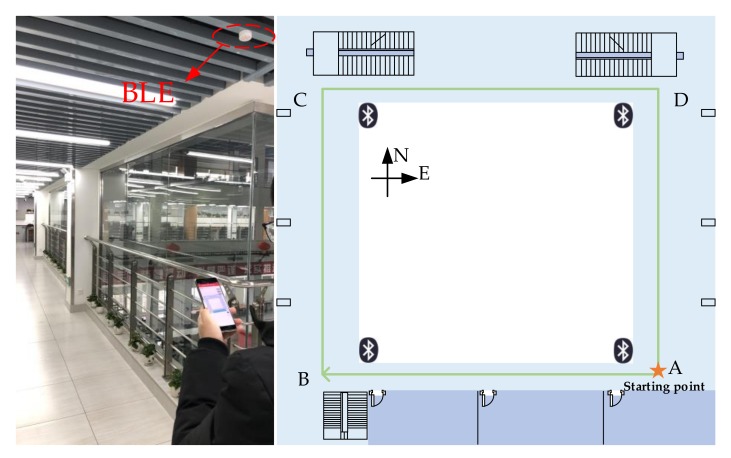
Large scale indoor test environment.

(2) Experiment 2: In a large-scale indoor environment with an area of about 25 × 23 m^2^, we performed a long-range positioning test. Following the same procedure as in Experiment 1, volunteers walked along the prescribed route A–B–C–D–A, the path length of each circle is about 70 m including four turning points, as shown in Figure 14. In order to compare the positioning performance more systematically, we installed Bluetooth low energy (BLE) at four locations in the test environment and implemented a commercially mature BLE calibration indoor positioning solution. The basic positioning principle of Bluetooth positioning is that in the offline stage, the experimenters deploy Bluetooth into the indoor environment and configure the location and MAC address of each Bluetooth and other information. In real-time positioning, when a user passes a Bluetooth beacon, the Bluetooth signal strength detected by the mobile phone generally exhibits a normally distributed change trend. Determine whether the cumulative error of calibration is by judging whether the signal strength value of the Bluetooth beacon is greater than the set threshold. The experimental comparison items in this part include original PDR positioning, BLE calibration positioning, and turning fusion calibration positioning.

**Figure 15:**
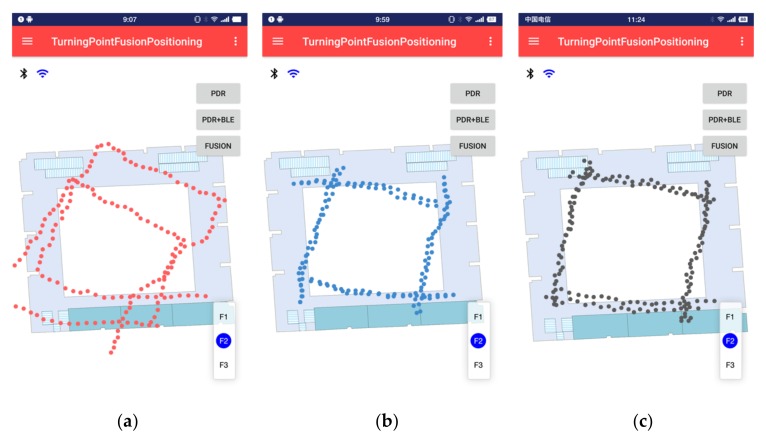
Positioning test results in a large-scale indoor environment: (**a**) Results of PDR positioning; (**b**) Results of Bluetooth low energy (BLE) calibration; and (**c**) Results of fusion calibration.

In this article, we have implemented three different positioning methods under the same application software. Testers can click different buttons to select different test items and follow the set path. The length of the total test path is about 140 m, and the positioning results are stored in the form of files in the root directory of the smartphone for evaluation of test accuracy. The walking trajectory of one of the testers is shown in Figure 15.

**Figure 16:**
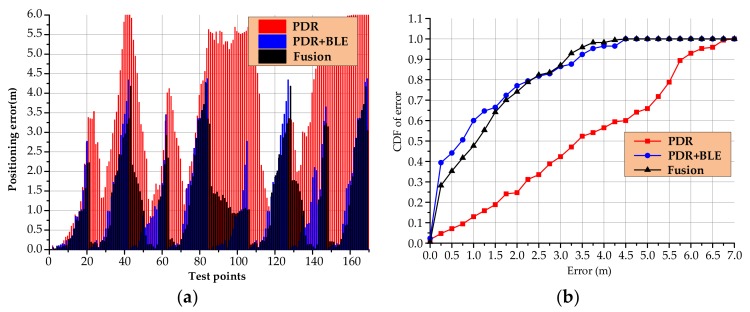
Statistical analysis of a large-scale indoor environment: (**a**) Linear distance error and (**b**) Cumulative error distribution function.

In Figure 15, the red marks are the PDR walking trajectories without calibration, the blue marks are the positioning results after BLE calibration and the black marks are the positioning results after fusion calibration we have proposed. It can be clearly seen that the schemes in Figure 15b,c can play the role of calibrating accumulated errors in a large-scale indoor environment. Specific positioning error statistics are shown in Figure 16.

It can be seen from Figure 16 that in a large-scale indoor environment, the original PDR positioning algorithm has a large cumulative error due to a long walking distance. The average positioning error is 3.51 m, the maximum positioning error is 6.78 m, and 65% of the positioning errors are less than 5.01 m. After BLE calibration, the average positioning error is 1.18 m, the maximum positioning error is 4.37 m, and 65% of the positioning errors are less than 1.49 m. The average positioning error of our proposed algorithm is 1.29 m, the maximum positioning error is 4.26 m, and 65% of the positioning errors are less than 1.50 m. However, it has to be said that the method proposed in this paper has the same positioning performance as the second method without the need to deploy additional equipment. In addition, in the second method, since the smartphone needs to continuously enable Bluetooth scanning during real-time positioning, the mobile phone generates heat and consumes a large amount of power. In summary, a conclusion can be drawn through the above experiments: the scheme we designed can calibrate the PDR algorithm and obtain a relatively good positioning effect, which verifies the feasibility of this lightweight indoor positioning solution. If higher positioning accuracy is needed, it may be necessary to explore more natural feature data that can be used for calibration. The specific statistical results of positioning accuracy are shown in Table 4.

**Figure 17:**
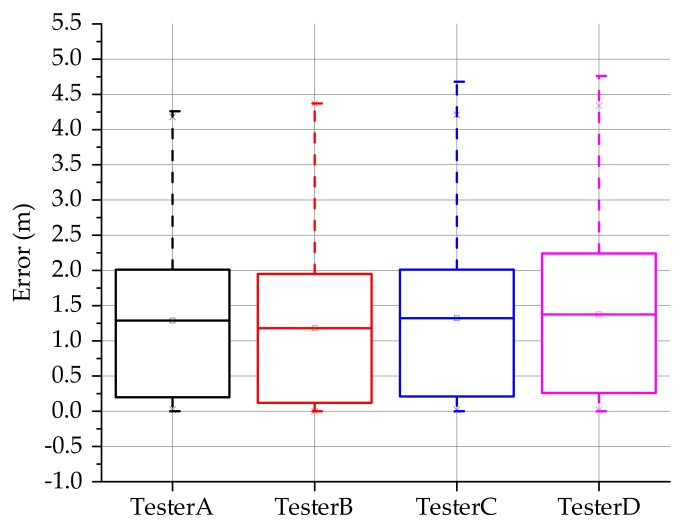
Adaptability of positioning model by different testers.

In order to verify the adaptability of the proposed positioning model to different users, we selected four testers, holding the same smartphone to walk along the route, and the test results are shown in Figure 17.

The box-plot is used to count and analyze the positioning results of different testers. The solid line inside each box-plot is the average value of the positioning error. By comparison, it is found that the average value of the positioning error of each user is about 1.5 m. The first quartile of different test results is less than 0.5 m. Therefore, it can be seen that the model has better adaptability to different users and can achieve similar positioning effects.

(3) Experiment 3: Smartphone adaptability test of indoor positioning software. Due to the different performance of MEMS sensors carried by different mobile phone manufacturers, it is likely that the algorithm proposed in this paper cannot achieve the ideal positioning effect. In order to verify the adaptability of the model on different smart terminals, we stored the model in different models of mobile phones (HUAWEI P20, HUAWEI Mate10, XIAOMI 8, and MEIZU 15). The configuration list of the mobile phone is shown in Table 5.

**Figure 18:**
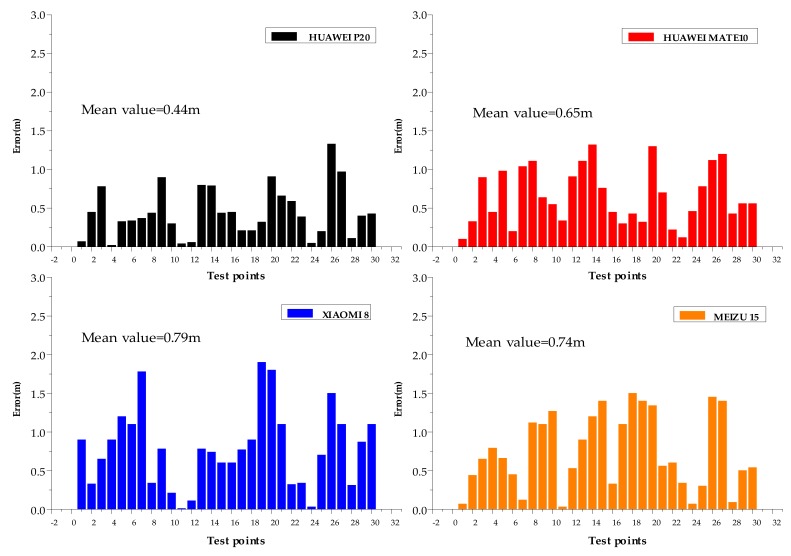
Adaptability of positioning model at different terminals.

The tester carried out experiments in the first environment with four smartphones, selected 30 measurement points in the test area, and analyzed the positioning accuracy. The experimental results are shown in Figure 18.

It can be seen from the experimental results that the average positioning accuracy of HUAWEI P20 is 0.44 m, and the positioning accuracy within 1 m is 97%; the average positioning accuracy of HUAWEI MATE 10 is 0.65 m, and the positioning accuracy within 1 m is 77%. The average positioning accuracy of XIAOMI 8 is 0.79 m, and the positioning accuracy within 1 m is 72%; the average positioning accuracy of MEIZU 15 is 0.74 m, and the positioning accuracy within 1 m is 70%. Although there are differences in the positioning accuracy of different receiving terminals, 70% of their positioning errors are less than 1 m. The system proposed in this paper has good adaptability, and can basically meet the positioning needs of most indoor environments, such as in large shopping malls or large conference venues, to find the rooms or shops you want to go to.

## 5. Discussion and Conclusions

An innovative indoor positioning system based on a smartphone MEMS sensor is proposed in this paper. First of all, this paper uses the accelerometer, gyroscope, and magnetometer to implement the PDR algorithm, which can provide users with a continuous relative position on the premise that the initial position is known. Then, in view of the accumulated error of the PDR algorithm due to long-term positioning, this paper designs a method that uses the environmental prior information to calibrate the accumulated error. In the offline phase, the training data set is constructed by collecting sensor data of pedestrians passing through the indoor turning point area, and then using the Tensorflow deep learning framework to build a sparse SDAE model in the python environment, the indoor turning point feature area detection model is obtained through training. In the online phase, after the turning point is identified using the trained turning point feature model, the PDR cumulative error is calibrated by a particle filter algorithm. Finally, this paper verifies the positioning performance of the proposed system through a large number of experiments in a real indoor environment.

During the experiment, there are still some disadvantages to this positioning system. For example, when there are fewer turning points in the indoor environment, the accuracy of the positioning system cannot be guaranteed. Moreover, when we use particle filtering to fuse turning point features and PDR positioning results, we found that if the number of particles is too large, the calculation efficiency of the mobile phone will be reduced, which cannot meet the fast indoor positioning. Therefore, in future research, we will continue to study more nonlinear filtering methods such as Extended Kalman Filtering or Unscented Kalman Filter [38] to improve the speed of position estimation. In order to achieve higher accuracy indoor positioning, we will continue to explore new natural features, such as lighting information, geomagnetic field information, and some signals-of-opportunity. By determining their measurement models, they are fused together to determine the user’s location to achieve a more stable and reliable low-cost indoor positioning system. Considering the randomness of the way users hold their smartphones, we will consider identifying more complex pedestrian activities in the next step.

## Figures and Tables

**Table 1:** Hyperparameters of the network model.

Hyperparameters	Values of Parameters
Hidden layers	256-256-128
Activation function	ReLU (Rectified Liner Unit)
Optimizer	Adam
learn rate	0.01
Weight decay	0.0005
Batch size	50
Epochs	300 (first DAE), 500 (second and third DAEs)

**Table 2:** The effect of sliding window size on classification.

Time Step	0.5 s	1 s	1.5 s	2 s	2.5 s	3 s	3.5 s	4 s
Recognition accuracy	61.2% (±0.08)	73.1% (±0.10)	80.2% (±0.11)	98.2% (±0.04)	93.5% (±0.11)	76.2% (±0.08)	31.2% (±0.10)	35.2% (±0.04)

**Table 3:** Statistics of positioning errors.

Algorithm	PDR	PDR-Calibrated
Mean Error (m)	0.83	0.14
95% Error (m)	2.56	0.35

**Table 4:** Statistics of positioning errors.

Algorithm	PDR	PDR + BLE	Fusion
Mean Error (m)	3.51	1.18	1.29
65% Error (m)	5.01	1.49	1.50
Maximum Error (m)	6.78	4.37	4.26

**Table 5:** Configuration list of different mobile phones.

No.	Version	OS	CPU (Hz)	RAM	ROM
1	HUAWEI P20	Android 8.1	2.4 G	6 G	64 G
2	HUAWEI Mate10	Android 7.0	2.4 G	4 G	64 G
3	XIAOMI 8	Android 8.0	2.8 G	6 G	64 G
4	MEIZU 15	Android 8.0	2.2 G	4 G	64 G
